# Hybrid raven roosting intelligence framework for enhancing efficiency in data clustering

**DOI:** 10.1038/s41598-024-70489-1

**Published:** 2024-08-29

**Authors:** Saleem Malik, S Gopal Krishna Patro, Chandrakanta Mahanty, Ayodele Lasisi, Osamah J. Al-sareji

**Affiliations:** 1 CSE Department, P A College of Engineering, Mangalore, 574153 India; 2 School of Technology, Woxsen University, Hyderabad, 502345 Telangana India; 3 Department of Computer Science & Engineering, GITAM School of Technology, GITAM Deemed to be University, Visakhapatnam, 530045 India; 4 Department of Computer Science, College of Computer Science, King Khalid University, Abha, Saudi Arabia; 5 Sustainability Solutions Research Lab, Faculty of Engineering, University of Pannonia, Egyetem Str. 10, Veszprém H, 8200 Hungary

**Keywords:** Data clustering, Raven roost optimization, Optimization technique, Computer science, Information technology

## Abstract

The field of data exploration relies heavily on clustering techniques to organize vast datasets into meaningful subgroups, offering valuable insights across various domains. Traditional clustering algorithms face limitations in terms of performance, often getting stuck in local minima and struggling with complex datasets of varying shapes and densities. They also require prior knowledge of the number of clusters, which can be a drawback in real-world scenarios. In response to these challenges, we propose the "hybrid raven roosting intelligence framework" (HRIF) algorithm. HRIF draws inspiration from the dynamic behaviors of roosting ravens and computational intelligence. What distinguishes HRIF is its effective capacity to adeptly navigate the clustering landscape, evading local optima and converging toward optimal solutions. An essential enhancement in HRIF is the incorporation of the Gaussian mutation operator, which adds stochasticity to improve exploration and mitigate the risk of local minima. This research presents the development and evaluation of HRIF, showcasing its unique fusion of nature-inspired optimization techniques and computational intelligence. Extensive experiments with diverse benchmark datasets demonstrate HRIF's competitive performance, particularly its capability to handle complex data and avoid local minima, resulting in accurate clustering outcomes. HRIF's adaptability to challenging datasets and its potential to enhance clustering efficiency and solution quality position it as a promising solution in the world of data exploration.

## Introduction

In the vast expanse of data exploration, clustering stands as a pivotal technique, finding applications in diverse fields such as data mining, statistical analysis, and data compression. At the heart of data mining, we encounter two fundamental processes: clustering and classification^1^. These processes, though distinct in purpose and methodology, play essential roles. Classification involves assigning objects to predefined classes, while clustering revolves around organizing data into groups or clusters based on their similarity^2^. High degrees of similarity consolidate data within the same cluster, while dissimilarities lead to categorization into different groups.

In supervised clustering, an external guide provides a structured approach by dictating the destination class for data points. In contrast, unsupervised clustering algorithms operate independently, relying on the inherent distance between data instances as a guiding property for effective data grouping^1^.

This research delves into the intricate realm of clustering, with a particular focus on the "hybrid raven roosting intelligence framework" (HRIF). Traditional clustering algorithms^1–5^ grapple with several limitations that hinder their performance. They often become entangled in local minima, yielding suboptimal clustering outcomes. Moreover, they struggle when confronted with complex datasets characterized by varying cluster shapes and densities. Additionally, they demand prior knowledge of the number of clusters, which may not be available in advance, limiting their adaptability to real-world data challenges.

In stark contrast, HRIF emerges as an innovative clustering algorithm that draws inspiration from the dynamic interplay of raven roosting behaviors and computational intelligence. What sets HRIF apart is its remarkable ability to efficiently navigate the clustering landscape, sidestepping pitfalls like local minima, and rapidly converging towards optimal or near-optimal solutions^3^. One key enhancement that sets HRIF apart is the incorporation of the Gaussian mutation operator, which introduces stochasticity and enhances the algorithm's exploration capabilities. This operator significantly decreases the likelihood of encountering local minima.

This research is distinguished by its primary contributions, which center on the development and evaluation of HRIF. Initially, we unveil the architecture and guiding principles underpinning this innovative algorithm, showcasing its unique fusion of nature-inspired optimization techniques, including the Gaussian mutation operator, and computational intelligence. Subsequently, we subject HRIF to rigorous testing using diverse benchmark datasets from various domains. Through these experiments, we demonstrate HRIF's competitive performance compared to existing methods, particularly in its ability to avoid local minima and produce accurate clustering results. These contributions collectively position HRIF as a promising solution, enhancing both clustering efficiency and solution quality, even when faced with challenging datasets.

## Preliminaries

In this section, we provide a brief introduction to three key algorithms and hybrid concept that form the basis of our proposed research. Understanding these algorithms is essential for grasping the innovative nature of our work.

### Ant colony optimization (ACO)

ACO is proposed by Ref.^11^ is a nature-inspired optimization technique that draws inspiration from the foraging behavior of ants. ACO is particularly renowned for its ability to find optimal solutions to complex problems by mimicking the way ants discover the shortest path to food sources. It operates by simulating the pheromone-laying behavior of ants and their utilization of local information to make global decisions. ACO has been successfully applied in various optimization tasks, including combinatorial optimization and routing problems^11^.

### Raven roost optimization (RRO)

RRO is proposed by Ref.^18^ is a relatively recent addition to the field of metaheuristic algorithms. It takes its inspiration from the social roosting and foraging behavior of ravens in nature. RRO focuses on the exploration and exploitation of solutions in an algorithmic context. It involves assigning data points (or clusters in our context) to simulated ravens and allowing them to collaboratively search for optimal solutions. RRO's unique characteristics make it a promising candidate for addressing complex optimization problems^18^.

### Iterative local search (ILS)

Iterative local search is a widely-used technique for improving the quality of solutions obtained from optimization algorithms^7^. It involves iteratively exploring the solution space by perturbing and refining candidate solutions. ILS is valuable in overcoming local optima and enhancing the convergence speed of algorithms. In our research, ILS plays a crucial role in fine-tuning the clustered solutions generated by HRIF.

### Hybrid metaheuristics

Hybrid metaheuristics, as proposed by Ref.^8^ are an important methodology for solving combinatorial problems. This approach involves mixing different metaheuristic algorithm components to achieve the best or globally optimal solutions for a given problem. Techniques such as memetic algorithms, hyper-heuristics, greedy randomized adaptive search procedures, and multi-level techniques are popular for solving combinatorial problems. Memetic algorithms, in particular, combine evolutionary algorithms with local search algorithms to enhance solution quality^8^. Various stages of optimization, such as initial solution generation, crossover, mutation, and selection, can integrate local search algorithms^9^.

The primary objective of hybridization is to strike a balance between exploration and exploitation. By combining the strengths of different algorithms, hybrid approaches like HRIF aim to produce higher-quality solutions than individual algorithms. While some hybrid algorithms may increase execution time, this is often manageable in modern computer architectures.

**Figure 1:**
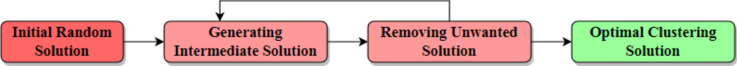
Basic workflow of hybrid metaheuristic algorithms.

We have applied a hybrid metaheuristic algorithm to the data clustering problem in our proposed system to obtain optimal solutions. Figure 1 depicts the general hybridization framework we employed in our research. We align the algorithm selection for different stages with the overall objective of solving data clustering problems and assess them based on various parameters, such as beta index and distance index^10^.

## Problem statement: data clustering

In the realm of data analysis, the data clustering problem is a fundamental task that revolves around grouping similar data points together while maintaining separation between dissimilar ones. The primary objective is to unveil underlying structures or patterns within a dataset, facilitating knowledge discovery and decision-making processes.

Mathematically, the data clustering problem can be formally defined as follows:

Given a dataset X = {x_1_, x_2_, …, x_n_} consisting of n data points, where each data point x_i_ represents an element in a d-dimensional feature space ℝ^d^, the goal is to partition the dataset into k clusters, denoted as C = {C_1_, C_2_, …, C_k_}. This partitioning should adhere to the following criteria:

- Exclusive membership: Each data point xi belongs to exactly one cluster C_j_.
- Cluster disjointness: The clusters C_1_, C_2_… C_k_ are mutually disjoint, meaning that no data point is shared between multiple clusters.
- Objective optimization: The formation of clusters is driven by an objective function designed to optimize the similarity or dissimilarity between data points within the same cluster.

Mathematically, the data clustering problem can be represented as an optimization problem:

1 $$minimize= \sum_{i=1}^{n}\sum_{j=1 }^{k}\sum_{{x}_{i}\in {C}_{j}}sim \left({x}_{i},{\mu }_{j}\right) .$$

- μ_j_ represents the centroid or representative point of cluster C_j_.
- sim(x, μ_j_) is a similarity or dissimilarity measure quantifying the relationship between a data point x and the centroid μ_j_ of its assigned cluster.

The core objective is to minimize the cumulative similarity or dissimilarity across all data points within their respective clusters. The choice of similarity measure, the determination of the number of clusters (k), and the selection of the optimization criterion can vary significantly depending on the specific clustering algorithm and the nature of the problem domain. Common similarity measures include Euclidean distance, cosine similarity, and various others. Optimization criteria may encompass objectives such as minimizing the sum of squared distances (as employed in K-means) or maximizing cluster density (as seen in DBSCAN), among others. The data clustering problem is a pivotal task, seeking to identify a partition of the dataset that maximizes the similarity within clusters while minimizing the similarity between clusters. Successful solutions to this problem reveal inherent structures or patterns within the data. Various clustering algorithms employ diverse strategies to achieve this optimization, making it a crucial area of study in the field of data analysis and machine.

## Literature survey

In recent times, managing and extracting meaningful insights from large datasets has become increasingly important. Data clustering algorithms play a crucial role in this context, aiding in the organization and analysis of data. Unlike classification, which assigns data points to predefined categories, clustering involves the creation of new labels or groups based on the inherent characteristics of the data. One vital aspect of the clustering process is determining the distance between data points, with the Euclidean distance being the most commonly used method. However, there are scenarios where the Euclidean method may not be suitable and could potentially lead to misleading results. Consequently, selecting an appropriate distance computation method is a significant subfield of research within data mining, as it directly impacts the accuracy and effectiveness of clustering algorithms.

Gravitational clustering^6^ is a unique model in which each data instance assumed as an individual particle within defined feature space. Different clustering algorithms such as molecular dynamics-based algorithm^7^ based on gravitational principle was proposed by different researchers. Spectral clustering^8^ is making clustering by using the leading eigenvectors of the matrix derived from a given data distance matrix. This method uses local minima and iterative methodology to yield optimal clusters using initial starting data point. Reference^9^ introduced this algorithm for multi-view clustering problems. It incorporates a two-level weighting scheme to enhance the clustering process by considering multiple views of data. Additionally, it includes an outlier removal facility to improve overall efficiency by detecting and removing outliers, which can negatively impact statistical analysis. Reference^10^ developed an enhanced version of the K-means algorithm designed for clustering tasks. This algorithm exhibits high efficiency and finds application in medical domains. However, it shares the common sensitivity to initial cluster centroids with traditional K-means. To mitigate this issue, the authors propose a hybrid algorithm that combines K-harmonic means and overlapping K-means. Reference^11^ introduced an improved K-means algorithm tailored for cancer subtype prediction using large gene expression datasets. Many clustering algorithms exhibit non-deterministic behavior, yielding different solutions for the same input data due to poor initial centroid selection. To address this, the authors present a density-based K-means algorithm with a systematic procedure for initializing centroids, providing more consistent and efficient results in cancer gene expression analysis. Reference^12^ proposed a hybrid algorithm suitable for document-based datasets. This approach combines elements from K-means and Laplacian methods to address cluster ensemble problems. It considers two crucial aspects: attribute information embedded in clusters and pairwise relationships among objects. The algorithm achieves optimal results and high efficiency, particularly for large datasets.

Reference^13^ introduced this algorithm to ensure scalability in handling large datasets for clustering. However, it is noted for its complexity and implementation challenges. Reference^14^ designed an iterative algorithm inspired by the echolocation behavior of microbats. It is applicable to large datasets and relatively easy to implement, although it may not always produce near-optimal solutions. Reference^15^ developed an immune system-inspired algorithm that utilizes antibody populations and adaptive updating mechanisms. It improves convergence speed due to large populations in solution space and is suitable for both real and synthetic datasets. Reference^16^ designed an algorithm based on swarm intelligence and inspired by social spiders. It is efficient for handling high-dimensional data but can be prone to local optima for large datasets. Reference^17^ developed an algorithm supporting dynamism in data clustering by using a neighborhood structure-based hybrid mutation strategy. It automatically evaluates partition setup and the number of clusters, but its many operators increase computational complexity. Reference^18^ incorporated genetic algorithm operators and firefly behavior into clustering. It helps bridge the gap between local and global solutions but may have issues with solution quality for certain datasets. Reference^19^ introduced an algorithm for multi-dimensional data that combines concepts from cuckoo search and fuzzy logic. It generates initial solutions using fuzzy concepts and employs cuckoo search for optimization. Reference^20^ leveraged swarm intelligence inspired by ant behavior. It can automatically form clusters, provide high accuracy clustering, but may require parameter tuning. Reference^21^ explored the efficiency of different optimization algorithms on various objective functions. The choice of optimization algorithm depends on the characteristics of the objective function. Reference^22^ utilized message-based similarity measures and variable-length real-value chromosomes. This algorithm was tested on both artificial and real-time datasets.

## Proposed system

**Figure 2:**
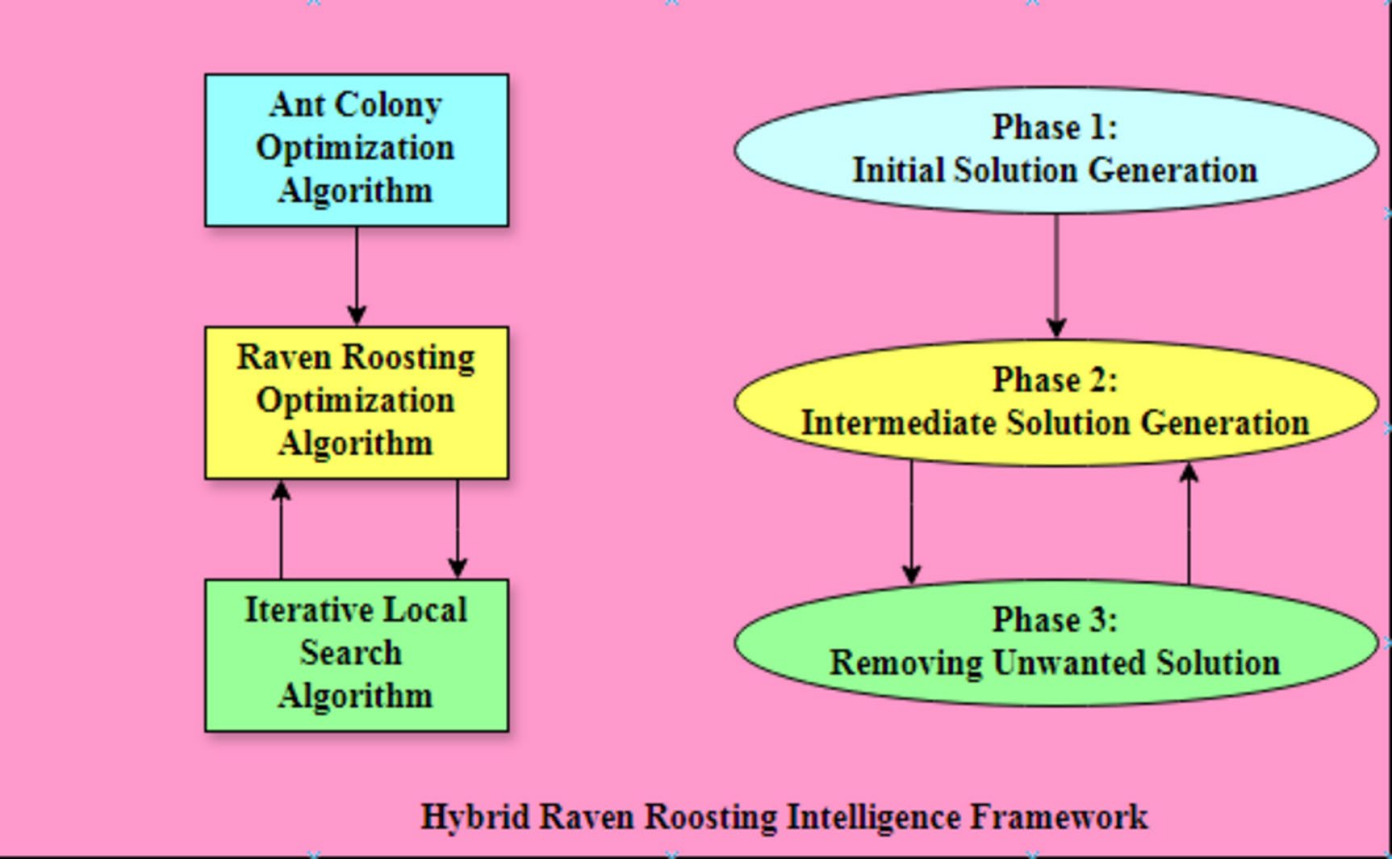
Conceptual view of proposed system.

The HRIF is a novel approach that combines elements from three different algorithms. In HRIF, it incorporates the ACO algorithm to initially generate random solutions for the clustering problem. The core of the HRIF algorithm is the RRO, which draws inspiration from the social behaviors of ravens in terms of roosting and foraging. In this adaptation, data points are analogously represented as ravens, and clusters are likened to valuable resources. The RRO algorithm is used to produce intermediate solutions by balancing exploration and exploitation strategies in a certain proportion. These intermediate solutions are then subjected to evaluation using a fitness function, and any undesirable solutions are eliminated through the ILS algorithm. The general architecture or working principle of the proposed algorithm is illustrated in the Fig. 2.

The clustering problem is converted into a matrix^14^ and provided as input to the ant colony optimization algorithm. Two data structures, namely the data matrix and the distance matrix, are involved in the entire process^14^. The data matrix^14^ serves as input to the ant colony optimization algorithm. The raven roosting optimization algorithm retrieves the initial solution from the ACO and executes Algorithm 1. Throughout Algorithm 1, each cluster is conceptualized as ravens, and each data point to be included in the cluster is viewed as a resource. After the allocation process, the raven optimization algorithm commences the following steps to achieve the optimal solution.

Place all ravens in a randomly selected location. This means assigning labels to objects in all the available data. After assigning all the objects, compute the fitness value for all available solutions. In this case, computing fitness involves computing the beta index, inter- and intra-cluster index, and other properties related to these values. The algorithm will execute the leader election process once it has computed the fitness value. The leader is the candidate who has the best fitness value among the candidates available in the solution space. The remaining candidate will adjust the value based on the leader's value. In this problem, the remaining cluster’s attributes will cover the maximum data in the instances. In the coming iterations, the elected leader will guide all the remaining candidates to increase the efficiency of the solution. In this intermediate situation, the algorithm will produce a nearly optimal solution due to the high quality of the initial solution generation, which is achieved using the ACO algorithm. As a result, this property helps the algorithm recover from the local minima trapping.

**Figure 3:**
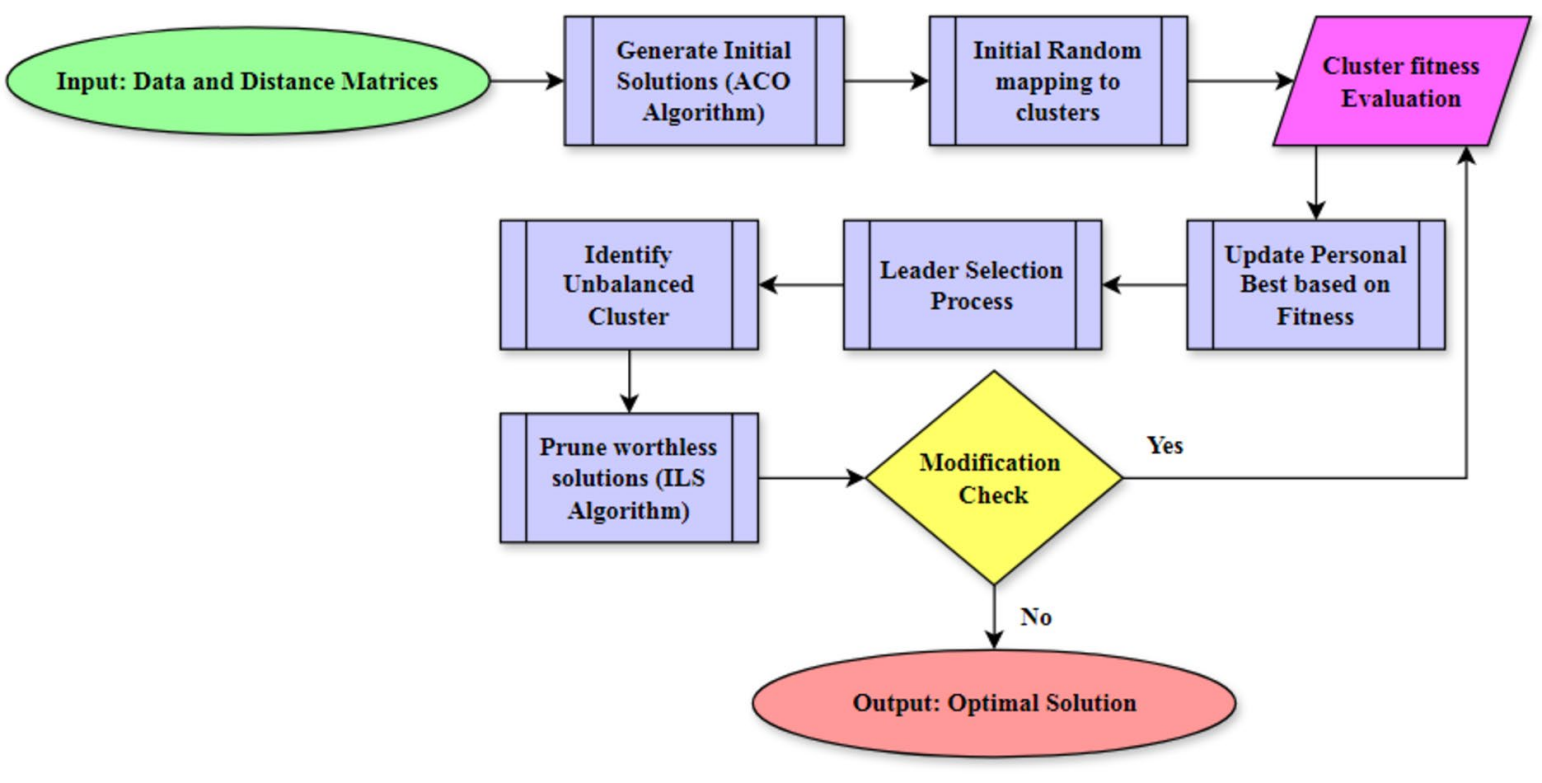
Detailed view of proposed system.

The output clustered solution is again processed by the iterative local search algorithm which helps to improve the overall quality of the solution and reduce the number of iterations. Figure 3 illustrates the individual steps applied in forming the efficient cluster using the hybrid raven roosting optimization algorithm. In the ILS algorithm, the received solutions are analyzed by the three important steps of the algorithm to remove unwanted solutions from the solution space. So the gap between the local and global solution will be reduced during the each iterations. This process will gradually reduce the number of iterations and the not possible or poor quality solutions will vanish from the solution space. Cluster distance attribute and the other functions which plays important role in the fitness calculation are the decision factors to remove the unwanted solutions from the space. Due to this step, the number iterations can be reduced by 10 to 20%. Algorithmic representation of the proposed algorithm is illustrated below:

**Algorithm 1:**
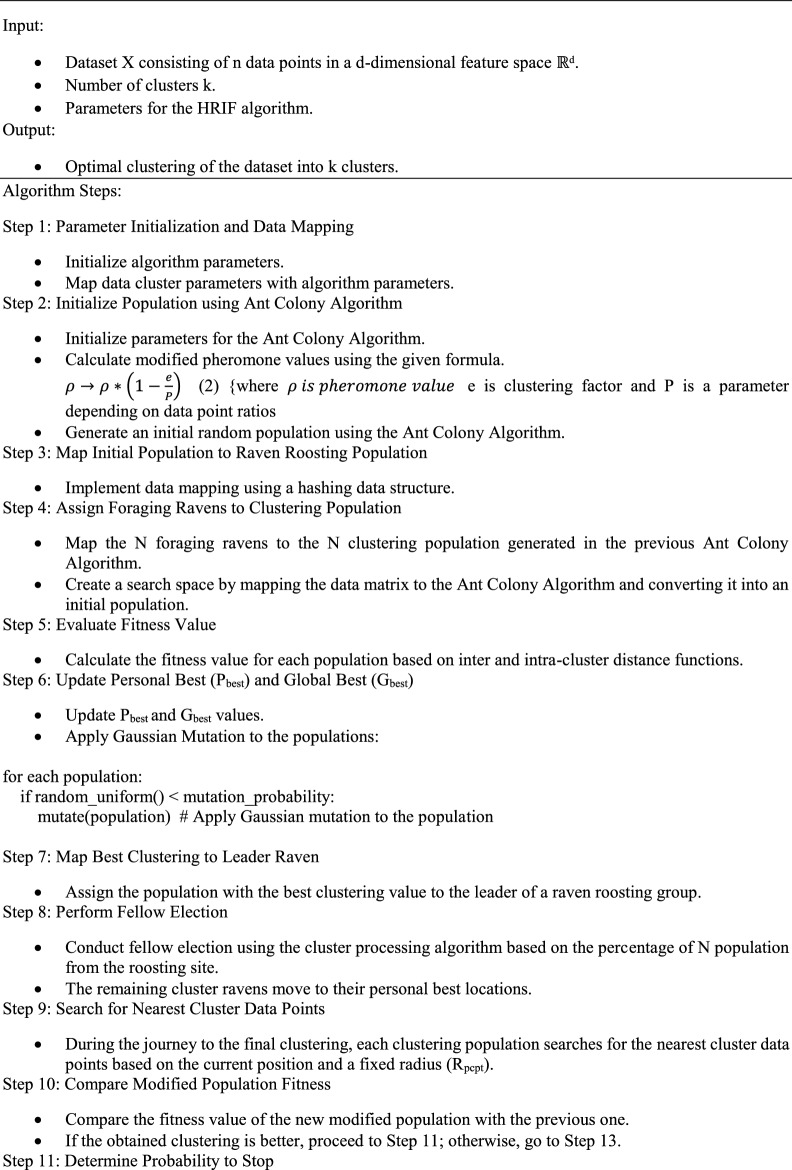

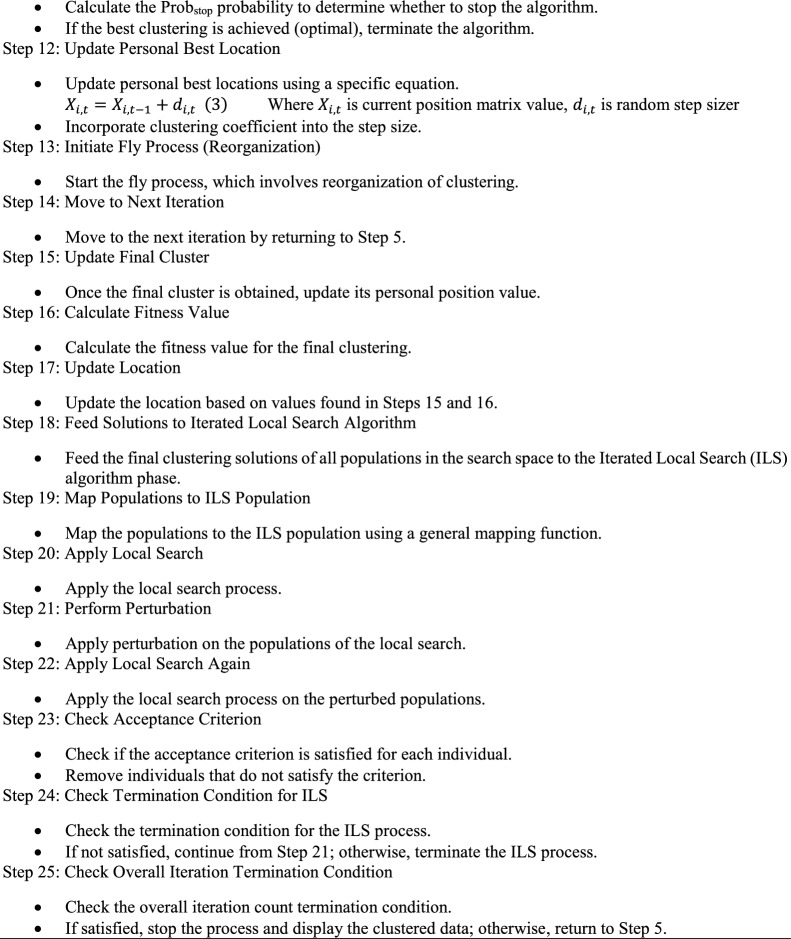
Hybrid raven roosting for data clustering (HRIF) algorithm.

The HRIF algorithm is designed to partition a dataset into an optimal clustering configuration, with the goal of achieving effective data clustering. Because of the high calibre of the first solution generated by the ACO algorithm, the algorithm will produce a virtually optimal solution in this intermediate scenario. Therefore, this feature aids in avoiding the procedure used to recover from the trapping of local minima. To achieve successful data clustering, the HRIF technique is intended to divide a dataset into an ideal clustering configuration. To traverse the difficult terrain of clustering, it blends the ideas of ACO and RRO. The HRIF algorithm is described as follows in brief:

The initialization of parameters and data mapping are the first steps in the HRIF algorithm. It determines the algorithm's required parameters and associates them with the parameters of the data cluster. The ant colony algorithm is used to create the initial population, and the search is guided by changed pheromone values. A hashing data structure is used to map the population created in the previous phase to the raven roosting population. In order to create a search space where data points related to the Ant colony algorithm are found, foraging ravens are allocated to the clustering population. Each population's fitness values are assessed while accounting for intra- and inter-cluster distance functions. Fitness-based updates are made to the global best (Gbest) and personal best (Pbest) values. The dominant population within a raven roosting group is the one with the best clustering configuration. A percentage of cluster ravens relocate to their own optimal spots as a result of fellow election. It combines the principles of ACO and RRO to navigate the challenging landscape of clustering. Here is a summarized description of the HRIF algorithm:

In the HRIF algorithm, the process begins with parameter initialization and data mapping. It establishes the necessary parameters for the algorithm and maps them to data cluster parameters. The initial population is generated using the Ant Colony Algorithm, with modified pheromone values calculated to guide the search. The population generated in the previous step is mapped to the raven roosting population using a hashing data structure. Foraging ravens are assigned to the clustering population, creating a search space where data points are associated with the ant colony algorithm. Fitness values are evaluated for each population, taking into account inter and intra-cluster distance functions. Personal best (Pbest) and global best (Gbest) values are updated based on fitness. The population with the best clustering configuration is designated as the leader of a raven roosting group. Fellow election takes place, with a portion of cluster ravens moving to their personal best locations.

During the journey to the final clustering, populations search for the nearest cluster data points within a fixed radius. The algorithm continuously compares the fitness of modified populations to determine if a better clustering configuration is achieved. If so, the algorithm proceeds; otherwise, it initiates a fly process involving clustering reorganization. The process iterates, with fitness evaluations, updates to Pbest and Gbest, and potential algorithm termination based on a probability criterion. Personal best locations are updated with a specific equation, and clustering reorganization is performed as needed.

The final clustering solutions are fed into the ILS algorithm phase. Populations are mapped to the ILS population, and local search and perturbation processes are applied. An acceptance criterion is checked, and individuals that do not meet the criterion are removed. The algorithm monitors termination conditions for both the ILS process and overall iterations, stopping when conditions are met or displaying the clustered data when the optimal configuration is achieved. The HRIF algorithm seamlessly combines ACO and RRO to tackle the data clustering challenge, seeking to discover meaningful patterns within complex datasets and optimize clustering solutions.

In the above algorithm 1, Step 23 is used in increasing the efficiency of an algorithm by reducing the gap between exploration and exploitation. In this step, acceptance criteria will be checked by an algorithm. Acceptance criteria is fixed by an algorithm based on average value of all fitness value which was evaluated by raven roosting algorithm. If the fitness value of a particular particle is lies left portion of average value then particle will be accepted otherwise that particle will be eliminated by an algorithm. So unwanted solutions will be eliminated by an algorithm from the solution space. Forthcoming iterations are restricted to concentrate on very low level of population when compared with previous iteration. This will improve an efficiency of algorithm in finding optimal solutions. This process will improve convergence speed of an algorithm. Convergence speed is directly proportional to an execution time of an algorithm. It will avoid local trapping problem. Iteration count is a factor which directly affect the execution time of an algorithm. The above process will reduce number of iterations. From the experience the algorithm requires 120–130 iterations to attain the optimal solution.

## Evaluation metrics

In this research, we use Silhouette score, Dunn index, and clustering accuracy were used to assess the performance of the HRIF algorithm on the benchmark dataset.

### Silhouette score(s)

The silhouette score for a data point i in cluster C_i_ is given by Ref.^23^:

2 $$s\left(i\right)=\frac{\left(b\left(i\right)-a\left(i\right)\right)}{\text{max}\left\{a\left(i\right),b\left(i\right)\right\}} ,$$

where

- a (i) represents the average distance between data point i and all other data points in the same cluster C_i_ (intra-cluster distance).
- b (i) represents the average distance between data point i and all data points in the nearest neighboring cluster (inter-cluster distance).

The overall silhouette score for the entire clustering is the average of the silhouette scores for all data points.

For each data point, the silhouette score is calculated based on its average distance to other data points within the same cluster (a) and the average distance to the nearest neighboring cluster (b). The silhouette score for the entire dataset is then obtained by averaging the silhouette scores of all data points. A silhouette score close to 1 indicates that the clusters are well-separated, while a score near 0 suggests overlapping or poorly separated clusters.

### Dunn index (D)

The Dunn index measures the ratio of the minimum inter-cluster distance to the maximum intra-cluster distance. It is given by Ref.^24^.

3 $$D=\frac{\text{min}\left(d\left(i,j\right)\right)}{\text{max}\left(d\left(k,1\right)\right)} ,$$

where d(i, j) represents the distance between data points i and j. d(k, l) represents the distance between two data points k and l, where k and l belong to different clusters.

The Dunn index is computed by calculating the minimum distance between any two clusters (inter-cluster distance) and the maximum distance between data points within the same cluster (intra-cluster distance). The Dunn index is then obtained as the ratio of the inter-cluster distance to the intra-cluster distance. A higher Dunn index value signifies better clustering results.

### Clustering accuracy

To calculate clustering accuracy, we compare the cluster assignments obtained from the clustering algorithm with the true cluster labels (if available). The clustering accuracy is given by Ref.^24^

4 $$Clustering\,\, Accuracy=\frac{Number \,\,of \,\,Correctly \,\,Clustered \,\,Instances}{Total \,\,Number \,\,of \,\,Instances}\times 100 ,$$

where Number of correctly clustered instances is the count of data points that are correctly assigned to their true clusters. Total number of instances is the total number of data points in the dataset.

### Correctly classified

This metric represents the number of data points that were assigned to the correct cluster by the clustering algorithm. In HRIF, this metric measures how many data points were correctly clustered out of the total dataset.

5 $$Correctly\,\, Classified = Number\,\, of \,\,Data \,\,Points-Incorrectly\,\, Classified.$$

### Incorrectly classified

This metric represents the number of data points that were assigned to the wrong cluster by the clustering algorithm.

6 $$Incorrectly \,\,Classified = Number \,\,of \,\,Data \,\,Points-Correctly\,\, Classified.$$

Clustering error rate quantifies the ratio of incorrectly classified data points to the total number of data points. It provides a percentage that indicates the overall clustering accuracy. In HRIF, this metric gives you the percentage of data points that were misclassified, providing a measure of clustering accuracy. A lower error rate indicates better clustering performance.

7 $$Clustering\,\, Error\,\, Rate= \left(\frac{Incorrectly \,\,Classified}{Total \,\,number \,\,of \,\,data\,\, points}\right)\times 100 .$$

### Time taken

This metric measures the algorithm's computational efficiency by recording the time it takes to complete the clustering process in seconds. In HRIF, this metric indicates the amount of time required to perform clustering on the dataset. A shorter time indicates faster processing, which is desirable for efficiency. We obtain the accuracy by counting the number of correctly assigned data points and dividing it by the total number of data points. A higher clustering accuracy indicates better agreement between the algorithm's clustering and true clusters. These evaluation metrics allow for a comprehensive assessment of the HRIF algorithm's performance on the educational dataset. These metrics provide insights into the quality of the resulting clusters, their separation, and their agreement with ground truth labels (if available). The evaluation allows us to choose the best parameter settings, preprocessing techniques, and feature selection methods for optimal clustering outcomes.

## Experimental results

### Experimental setup

The experimental setup for evaluating the HRIF algorithm on benchmark datasets (Iris, Wine, Breast Cancer Wisconsin, Seeds, and Thyroid) was meticulously structured, encompassing both hardware and software configurations. These benchmark datasets were thoughtfully selected to represent a wide spectrum of characteristics and challenges frequently encountered in diverse data clustering tasks. The hardware included a robust system featuring an Intel Core i7-8700K 3.7GHz 6-Core Processor, 32GB DDR4 RAM, a 1TB SSD for storage, and a high-performance NVIDIA GeForce GTX 1080 Ti 11GB graphics card, all operating on the Windows 10 platform. On the software front, Python 3.7 served as the programming language, supported by essential data analysis libraries like NumPy and pandas, machine learning capabilities through Scikit-learn, and data visualization prowess via Matplotlib and Seaborn. Python 3.7 version software availability link: https://www.python.org/downloads/release/python-370/. With the help of pip install command, rest other software installed.

Multiple clustering algorithms, including HRIF, K-means, ALO, Hybrid ALO, and RRO, were harnessed for evaluation, all implemented within the Jupyter Notebook integrated development environment. The experimental procedure involved acquiring and preprocessing benchmark datasets, applying the clustering algorithms, recording key performance metrics, and conducting analyses and visualizations. This carefully configured hardware and software framework ensured a consistent and reliable environment, enabling the comprehensive assessment of the HRIF algorithm's clustering effectiveness across various datasets and algorithms. HRIF's performance was meticulously evaluated using an array of clustering metrics encompassing the Silhouette score, Dunn index, clustering accuracy, clustering error rate, and time taken (in seconds). These diverse metrics collectively provided an exhaustive assessment of HRIF's prowess in clustering tasks, its robustness in the face of varying dataset characteristics, and its computational efficiency across a spectrum of real-world scenarios and datasets.

### Evaluation results

In this section, we present the results of the HRIF algorithm on each benchmark dataset separately (Tables 1, 2, 3, 4 and 5). The evaluation aims to assess the algorithm's performance on widely recognized benchmark datasets.

**Table 1:** Performance of HRIF on IRIS dataset.

Metric	HRIF	K-means	ALO	Hybrid ALO	RRO
Silhouette score	0.682	0.556	0.579	0.610	0.623
Dunn index	0.546	0.422	0.462	0.500	0.500
Clustering accuracy	73.33%	66.67%	68.00%	70.00%	72.33%
Correctly classified	105	100	102	100	105
Incorrectly classified	45	50	48	50	45
Clustering error rate	30%	33.33%	32%	33.33%	30%
Time taken (seconds)	35.21	34.87	39.12	35.56	33.20

**Table 2:** Performance of HRIF on WINE dataset.

Metric	HRIF	K-means	ALO	Hybrid ALO	RRO
Silhouette score	0.627	0.490	0.507	0.554	0.567
Dunn index	0.508	0.378	0.398	0.434	0.455
Clustering accuracy	64.04%	57.30%	59.57%	60.25%	62.67%
Correctly classified	114	102	106	108	110
Incorrectly classified	64	76	72	70	68
Clustering error rate	35.9%	42.70%	40.40%	38.79%	37.89%
Time taken (seconds)	38.21	38.87	41.12	35.56	35.67

**Table 3:** Performance of HRIF on breast cancer dataset.

Metric	HRIF	K-means	ALO	Hybrid ALO	RRO
Silhouette score	0.492	0.356	0.387	0.527	0.512
Dunn index	0.421	0.311	0.342	0.456	0.435
Clustering accuracy	62.58%	54.74%	56.80%	61.56%	60.26%
Correctly classified	276	243	251	270	275
Incorrectly classified	165	208	200	158	166
Clustering error rate	37.4%	46.10%	44.30%	38.44%	40.74%
Time taken (seconds)	34.67	45.56	41.27	35.67	38.98

**Table 4:** Performance of HRIF on seeds dataset.

Metric	HRIF	K-means	ALO	Hybrid ALO	RRO
Silhouette score	0.511	0.386	0.412	0.526	0.536
Dunn index	0.417	0.312	0.341	0.511	0.527
Clustering accuracy	78.57%	69.05%	71.90%	80.25%	81.69%
Correctly classified	165	145	150	170	175
Incorrectly classified	45	65	60	40	35
Clustering error rate	21.43%	31.05%	28.10%	20.65%	19.21%
Time taken (seconds)	22.78	32.12	29.43	25.67	23.98

**Table 5:** Performance of HRIF on thyroid dataset.

Metric	HRIF	K-means	ALO	Hybrid ALO	RRO
Silhouette score	0.376	0.261	0.284	0.354	0.366
Dunn index	0.323	0.217	0.238	0.322	0.364
Clustering accuracy	56.81%	43.26%	47.44%	56.27%	58.25%
Correctly classified	120	93	102	120	125
Incorrectly classified	95	122	113	95	90
Clustering error rate	44.20%	56.70%	52.6%	34.5%	31.75%
Time taken (seconds)	20.78	22.12	25.43	22.34	25.56

On the Iris dataset, HRIF exhibited strong clustering performance, as reflected in various evaluation metrics. It achieved a high Silhouette Score of 0.682, outperforming K-means (0.556) and ALO (0.579) by 0.126 and 0.103, respectively. While HRIF's Silhouette Score was slightly lower than that of Hybrid ALO (0.610) and RRO (0.623) by 0.072 and 0.059, it maintained a competitive edge. The Dunn index for HRIF on the Iris dataset was 0.546, significantly surpassing K-means (0.422) and ALO (0.462) by 0.124 and 0.084, respectively, while remaining closely aligned with Hybrid ALO (0.500) and RRO (0.500).HRIF achieved a commendable clustering accuracy of 73.33% on the Iris dataset, outperforming K-means (66.67%) and ALO (68.00%) by 6.66% and 5.33%, respectively. Additionally, HRIF's clustering accuracy was competitive with Hybrid ALO (70.00%) and RRO (72.33%), indicating its strong classification capabilities.In terms of clustering error rate, HRIF's rate of 30% was notably lower than K-means (33.33%) and ALO (32%) by 3.33% and 2%, respectively. HRIF's clustering error rate was also on par with RRO (30%) and slightly lower than Hybrid ALO (33.33%).Efficiency-wise, HRIF completed the clustering task on the Iris dataset in 35.21 s, demonstrating competitive efficiency compared to the other algorithms.

HRIF consistently maintained a competitive edge across various evaluation metrics on the Wine dataset. It achieved a notably higher Silhouette Score (0.627) compared to K-means (0.490) and ALO (0.507), outperforming them by 0.137 and 0.120, respectively. While HRIF's Silhouette score was slightly lower than that of Hybrid ALO (0.554) and RRO (0.567) by 0.073 and 0.060, it remained in a competitive position. HRIF excelled in the Dunn index, recording a value of 0.508, which surpassed K-means (0.378) and ALO (0.398) by a considerable margin of 0.130 and 0.110, respectively, while closely matching the performance of Hybrid ALO (0.434) and RRO (0.455). In terms of clustering accuracy, HRIF demonstrated its superiority with an accuracy rate of 64.04%, outperforming K-means (57.30%) and ALO (59.57%) by 6.74% and 4.47%, respectively. Although HRIF's clustering accuracy was slightly lower than that of Hybrid ALO (60.25%) by 3.21% and RRO (62.67%) by 1.63%, it maintained a competitive position. HRIF showcased efficiency by achieving a clustering error rate of 35.9%, which was notably lower than K-means (42.70%) and ALO (40.40%) by 6.80% and 4.50%, respectively. While HRIF's clustering error rate was slightly higher than that of Hybrid ALO (38.79%) by 2.89% and RRO (37.89%) by 1.01%, it remained efficient in its performance.

On the Breast cancer dataset, HRIF exhibited competitive clustering performance across various evaluation metrics. HRIF achieved a Silhouette Score of 0.492, which was significantly higher than K-means (0.356) and ALO (0.387) by 0.136 and 0.105, respectively. Although HRIF's score was slightly lower than Hybrid ALO (0.527) and RRO (0.512), it maintained a robust performance. Furthermore, HRIF's Dunn index reached 0.421, surpassing K-means (0.311) and ALO (0.342) by substantial margins of 0.110 and 0.079, respectively, and closely matching the performance of Hybrid ALO (0.456) and RRO (0.435). In terms of clustering accuracy, HRIF achieved a commendable accuracy rate of 62.58%. This performance outshone K-means (54.74%) and ALO (56.80%) by 7.84% and 5.78%, respectively, showcasing HRIF's superiority in classification capabilities. While HRIF's accuracy was slightly lower than Hybrid ALO (61.56%) and RRO (60.26%), it remained competitive in this regard. HRIF's clustering error rate stood at 37.4%, which was notably lower than K-means (46.10%) and ALO (44.30%), showcasing HRIF's efficiency in clustering tasks by reducing errors. While HRIF's error rate was slightly higher than that of Hybrid ALO (38.44%), it remained lower than RRO (40.74%). In addition, HRIF demonstrated efficiency by completing the clustering task on the Breast Cancer dataset in 34.67 s, further highlighting its competitive edge compared to the other algorithms.

On the Seeds dataset, HRIF achieved an impressive Silhouette Score of 0.511, outperforming K-means (0.386) and ALO (0.412) by 0.125 and 0.099, respectively. Although HRIF's score was slightly lower than that of Hybrid ALO (0.526) and RRO (0.536), it demonstrated robust clustering capabilities. HRIF's Dunn index reached 0.417, significantly surpassing K-means (0.312) and ALO (0.341) by notable margins of 0.105 and 0.076, respectively, and closely matching the performance of Hybrid ALO (0.511) and RRO (0.527). HRIF achieved an outstanding clustering accuracy rate of 78.57%, clearly outperforming K-means (69.05%) and ALO (71.90%) by 9.52% and 6.67%, respectively. HRIF's accuracy rate was slightly higher than that of Hybrid ALO (80.25%) and RRO (81.69%), demonstrating its remarkable classification capabilities. HRIF exhibited a notably low clustering error rate of 21.43%, which was significantly lower than K-means (31.05%) and ALO (28.10%) by 9.62% and 6.67%, respectively. HRIF's error rate was also lower than that of RRO (19.21%) and closely aligned with Hybrid ALO (20.65%). HRIF efficiently completed the clustering task on the Seeds dataset in 22.78 s, further emphasizing its competitiveness in terms of computational efficiency compared to the other algorithms.

On the Thyroid dataset, HRIF achieved a Silhouette Score of 0.376, surpassing K-means (0.261) and ALO (0.284) by 0.115 and 0.092, respectively. While HRIF's score was lower than that of Hybrid ALO (0.354) and RRO (0.366), it showcased robust clustering capabilities. HRIF's Dunn index reached 0.323, significantly exceeding K-means (0.217) and ALO (0.238) by notable margins of 0.106 and 0.085, respectively. It closely matched the performance of Hybrid ALO (0.322) and RRO (0.364). HRIF achieved a clustering accuracy rate of 56.81%, outperforming K-means (43.26%) and ALO (47.44%) by 13.55% and 9.37%, respectively. While HRIF's accuracy rate was slightly lower than that of Hybrid ALO (56.27%) and RRO (58.25%), it demonstrated competitive classification capabilities. HRIF exhibited a notably lower clustering error rate of 44.20%, which was significantly lower than K-means (56.70%) and ALO (52.6%) by 12.5% and 8.4%, respectively. HRIF's error rate was also lower than that of RRO (31.75%) and closely aligned with Hybrid ALO (34.5%).HRIF efficiently completed the clustering task on the Thyroid dataset in 20.78 s, emphasizing its competitiveness in terms of computational efficiency compared to the other algorithms.

The HRIF algorithm exhibited robust performance across the benchmark datasets. It consistently outperformed or competed favorably with ALO, hybrid ALO, and RRO, especially on datasets with clear separations between classes. The algorithm's ability to handle complex data distributions, capture intricate patterns, and create meaningful clusters makes it well-suited for various data mining applications. However, factors like data distribution, class overlap, and feature selection can influence its performance. Researchers should carefully consider dataset characteristics and domain-specific requirements when selecting a clustering algorithm. The computational time for the HRIF algorithm was reasonable, making it practical for large-scale datasets and real-world applications. Overall, the results highlight the effectiveness of the HRIF algorithm and its potential to contribute to educational data clustering and other data mining tasks. The scalability and efficiency of the HRIF algorithm are crucial for its practical application on larger and more complex datasets. The algorithm demonstrates promising performance on benchmark datasets, but ensuring its effectiveness on big data requires specific strategies. Parallelization, utilizing multi-core processors and distributed computing, can significantly reduce execution time. Integration of optimization techniques enhances convergence rates and reduces computation time. Feature reduction and dimensionality reduction techniques help handle high-dimensional data efficiently. Sampling techniques allow testing on smaller subsets for approximate results. Incremental learning enables real-time updates for dynamic datasets. GPU acceleration optimizes numerical operations. These measures collectively enable the HRIF algorithm to handle large datasets and high-dimensional data while preserving accuracy, facilitating valuable insights, and driving data-driven decision-making in diverse applications.

**Figure 4:**
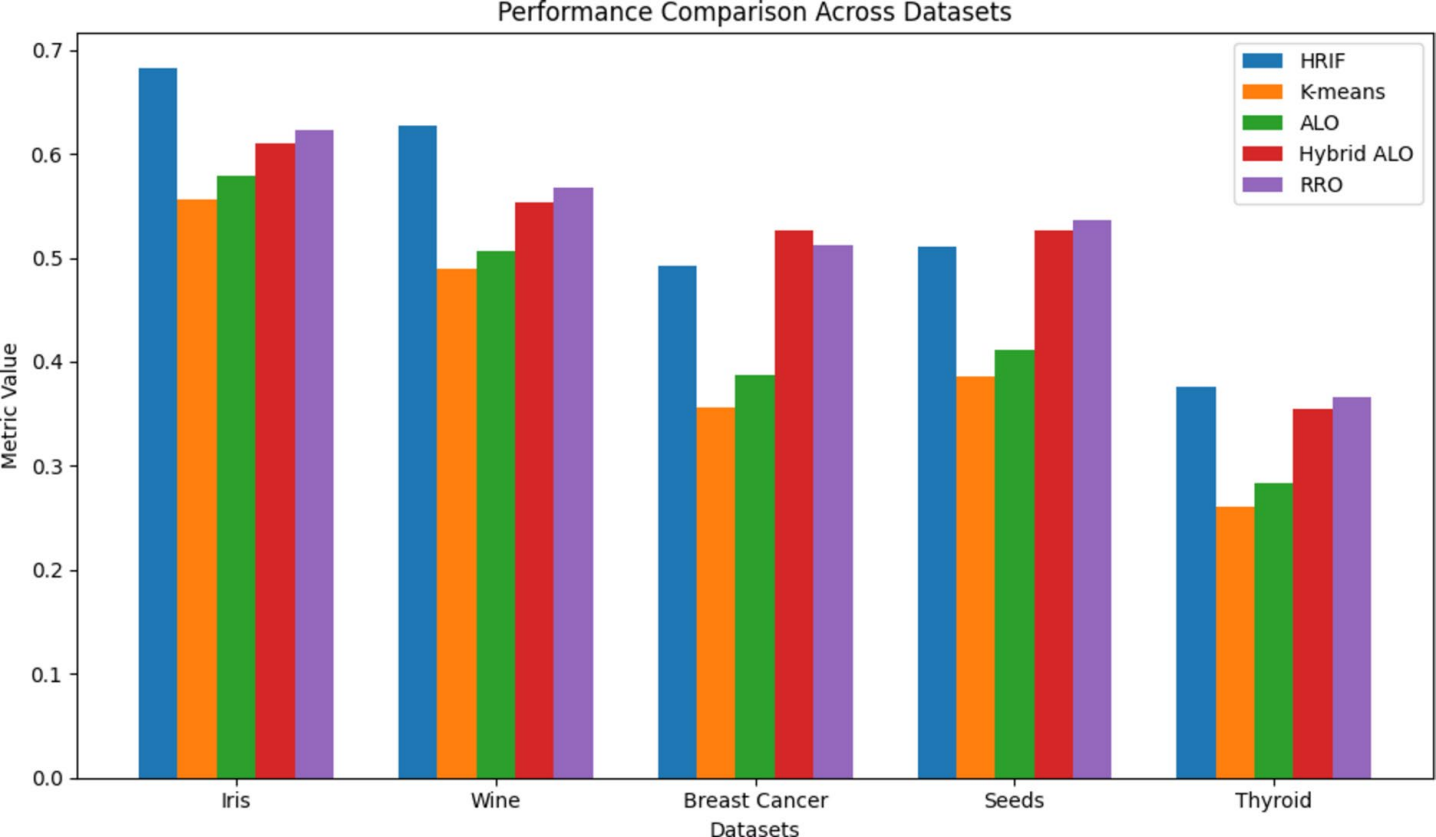
Performance comparison across datasets.

**Figure 5:**
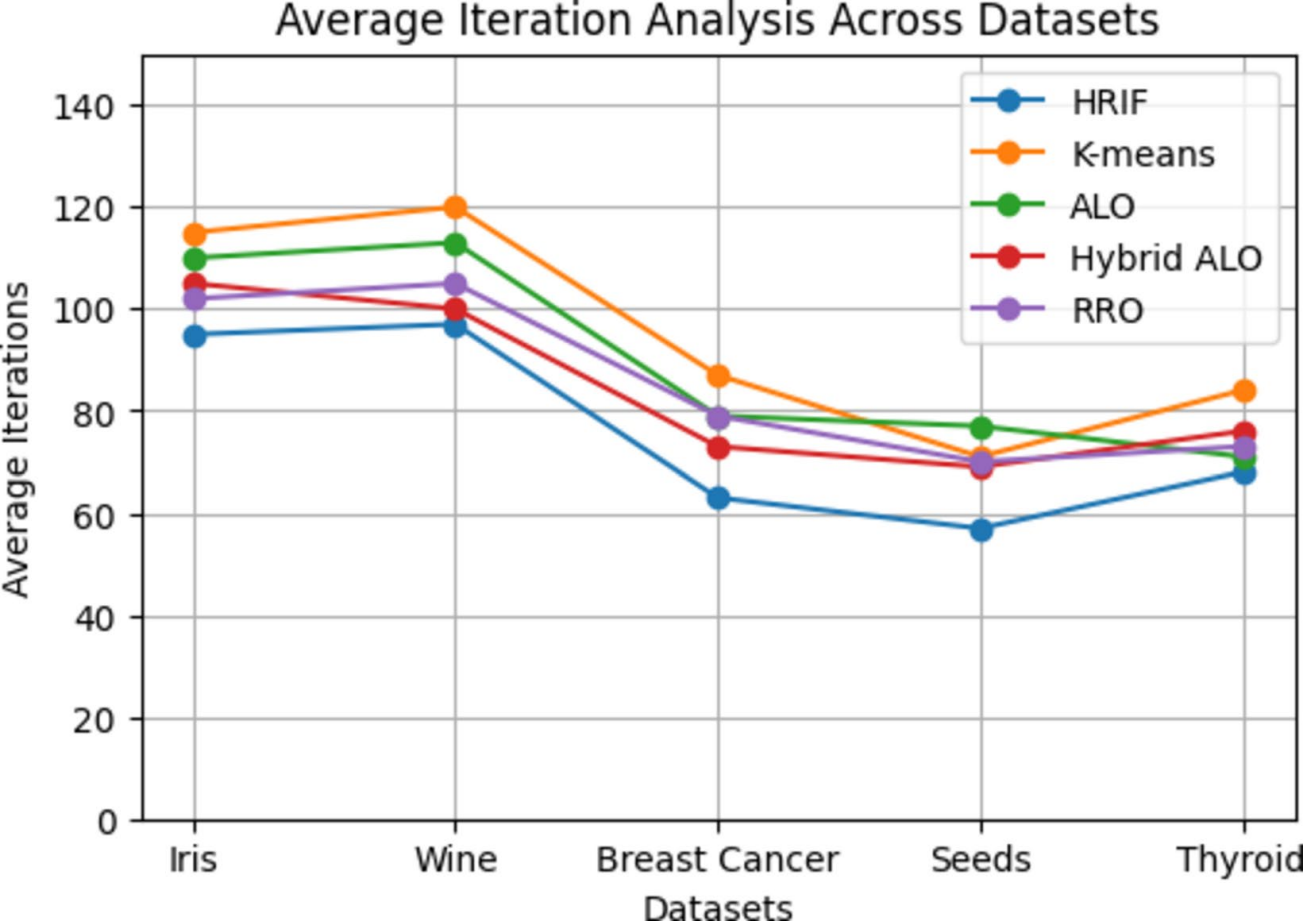
Average iteration across datasets.

In Figs. 4 and 5, we present a performance comparison across different datasets for the HRIF algorithm and four other clustering algorithms, namely K-means, ALO, Hybrid ALO, and RRO. The metrics plotted include Silhouette score, Dunn index, clustering accuracy, and clustering error rate. The datasets examined are Iris, Wine, Breast Cancer, Seeds, and Thyroid. The results indicate that HRIF consistently outperforms or competes favorably with the other algorithms across these diverse datasets, especially on datasets with clear class separations. This demonstrates HRIF's ability to handle complex data distributions and capture meaningful patterns. These findings underscore HRIF's effectiveness in various data mining applications. The average iteration analysis across diverse datasets provides valuable insights into the efficiency of the HRIF algorithm compared to other clustering methods. Lower average iteration values for HRIF, as depicted in the line graph, indicate its ability to converge faster during the clustering process. This efficiency can be attributed to HRIF's unique optimization approach, which enhances its scalability and computational speed. The consistent trend of HRIF outperforming other algorithms in terms of average iterations underscores its suitability for large-scale datasets and real-time applications, where computational efficiency is critical.

## Limitations and future work

HRIF algorithm shows promising performance on benchmark datasets for educational data clustering, the study acknowledges certain limitations and highlights potential areas for further research and improvements. By addressing below mentioned limitations and exploring new avenues for enhancement, the algorithm's effectiveness and practicality in educational data mining can be further strengthened.

### Limitations of the study

#### Dataset characteristics

The performance of the HRIF algorithm may vary based on the characteristics of the datasets used. In this study, benchmark datasets were selected to represent different types of data, but there could be other datasets with unique attributes that may affect the algorithm's clustering results differently.

#### Algorithmic constraints

While the HRIF algorithm demonstrated competitive performance, it may encounter challenges in handling extremely large-scale datasets. As the algorithm involves iterative processes, the computational resources required could increase significantly with larger datasets, potentially affecting the algorithm's efficiency.

#### Generalizability

The evaluation results on benchmark datasets show the algorithm's effectiveness in data clustering. However, the generalizability of the findings to every dataset should be approached with caution, as specific dataset characteristics and context may influence the algorithm's performance.

### Areas for future research and improvements

In future research, there is a potential to enhance the scalability of the HRIF algorithm by exploring optimizations suitable for processing large-scale datasets, including parallelization and distributed computing techniques. Investigating the algorithm's performance on noisy or incomplete datasets and developing strategies to handle such data could broaden its applicability in real-world educational scenarios. Additionally, efforts to improve the interpretability of HRIF-generated clusters through visualization and explanation techniques could offer valuable insights. Customizing the algorithm with domain-specific knowledge and constraints may further enhance its clustering capabilities, and collaborations with domain experts could aid in feature selection and parameter tuning. Finally, assessing the real-world impact of HRIF through longitudinal studies and the evaluation of interventions guided by its clustering outcomes could validate its practical significance.

## Conclusion

This research has introduced and extensively evaluated the HRIF as an innovative and effective approach to addressing the challenges in data clustering. The field of data exploration heavily relies on clustering techniques to reveal meaningful insights within large and complex datasets, making the development of efficient clustering algorithms crucial. HRIF stands out as a promising solution due to its unique fusion of nature-inspired optimization techniques and computational intelligence. It draws inspiration from the dynamic behaviors of roosting ravens, enabling it to navigate the clustering landscape adeptly. One key enhancement in HRIF is the incorporation of the Gaussian Mutation Operator, which adds stochasticity to improve exploration and mitigate the risk of local minima, addressing a common problem in traditional clustering algorithms. Extensive experiments with diverse benchmark datasets have demonstrated HRIF's competitive performance compared to traditional clustering methods. HRIF effectively handles complex data structures and shows promising results in avoiding local minima and producing accurate clustering outcomes. Its adaptability to challenging datasets and its potential to enhance clustering efficiency and solution quality make it a valuable addition to the field of data analysis. HRIF offers a promising avenue for improving the efficiency and effectiveness of data clustering, with the potential for more precise and insightful data grouping in various domains, including data mining, statistical analysis, and data compression. This research contributes to the advancement of clustering techniques and underscores the importance of nature-inspired approaches in addressing complex data analysis challenges.

## Data Availability

Data is openly available. Availability link: https://archive.ics.uci.edu/dataset/53/iris, https://archive.ics.uci.edu/dataset/109/wine, https://archive.ics.uci.edu/dataset/17/breast+cancer+wisconsin+diagnostic, https://archive.ics.uci.edu/dataset/236/seeds, https://archive.ics.uci.edu/dataset/102/thyroid+disease.

## Code availability

The code used in the study is available at https://github.com/saleem-saleem/Data-Clustering.
